# Photothermal effects of CuS-BSA nanoparticles on H22 hepatoma-bearing mice

**DOI:** 10.3389/fphar.2022.1029986

**Published:** 2022-10-12

**Authors:** Xinyu Dun, Shuliang Liu, Nan Ge, Meng Liu, Ming Li, Jun Zhang, Hongxu Bao, Benying Li, Hua Zhang, Lianhua Cui

**Affiliations:** ^1^ Department of Toxicology, School of Public Health, Qingdao University, Qingdao, China; ^2^ Weihai Center for Disease Control and Prevention, Weihai, Shandong, China; ^3^ Department of Occupational Medicine, The Affiliated Qingdao Central Hospital of Qingdao University, The Second Affiliated Hospital of Medical College of Qingdao University, Qingdao, China; ^4^ Collaborative Innovation Center for Nanomaterials and Devices, College of Physics, Qingdao University, Qingdao, China

**Keywords:** CuS-BSA NPs, photothermal therapy, hepatocarcinoma, *in vivo*, apoptosis

## Abstract

The objective of this study was to evaluate the *in vivo* application and photothermal ablation effects and mechanism of copper sulfide nanoparticles (CuS NPs) in hepatocellular carcinoma (HCC). Sheet-like CuS-BSA NPs with a particle size of 30 nm were synthesized using bovine serum albumin (BSA) as a biological modifier, and were physically characterized. To provide a reference range for the biosafety dose of CuS-BSA NPs, 36 male Kunming mice were randomly assigned into six groups. Different one-time doses of CuS-BSA NPs were injected *via* tail vein injection, and the potential damages of liver, kidney and spleen were observed 14 days later. To evaluate the *in vivo* photothermal effect of CuS-BSA NPs, 48 male Kunming mice were used to establish the H22 hepatoma-bearing mouse model and were randomly assigned into six groups. CuS-BSA NPs (600 μg/kg) were injected *via* tail vein or intratumoral injection. Irradiations were performed 30 min after injection, with a 980 nm near-infrared laser (2.0 W/cm^2^) for 10 min once a week for 3 weeks. The results indicated that the CuS-BSA NPs had good dispersibility in three different solvents and had a strong absorption peak at 980 nm. The heating curves demonstrated that the photothermal effects of CuS-BSA NPs aqueous solution exhibited concentration dependence and power density dependence. In the *in vivo* experiment, when the doses of CuS-BSA NPs were in the range of 1800–7,200 μg/kg, the thymus index and spleen index of mice were not significantly different from those of the control group, and the structures of liver, kidney and spleen were intact without remarkable pathological changes. A lower dose of CuS-BSA NPs (600 μg/kg) could effectively inhibit tumor growth in H22 hepatoma-bearing mice at 980 nm NIR. Moreover, under the near-infrared laser irradiation, both in the tail vein injection group and the intratumoral injection group, a large area of necrosis in the tumor tissue, as well as the up-regulation of apoptotic proteins including cleaved caspase-3 and cleaved caspase-9 were observed. CuS-BSA NPs are promising photothermal agents in the photothermal therapy of cancer.

## Introduction

Hepatocellular carcinoma (HCC) is the sixth most common cancer and the third leading cause of cancer-related deaths globally in the global cancer burden in 2020 (Sung et al., 2021). Surgery is a curative treatment with a 5-year survival rate of over 50%, but only in patients with early-stage HCC (Kwon et al., 2021). Less than 20% of patients with HCC are eligible for undergoing surgical treatment because of the shortage of donor livers, hepatic insufficiency after hepatectomy, and unsuitability for surgery for advanced HCC(Clark et al., 2005; Davila et al., 2012; Moris et al., 2018). Severe toxic and side effects of chemoradiotherapy on normal human tissue caused by the nonspecific targeting of the tumor tissue limit its application. Moreover, most chemotherapeutic drugs have poor hydrophilicity and can develop resistance, resulting in unsatisfactory treatment outcomes (Xie et al., 2016; Sun et al., 2017).

The current situation of cancer treatment has prompted the emergence of a series of new treatment methods. Among numerous alternative therapies, near-infrared (NIR, *λ* = 700–1,000 nm) laser-induced photothermal therapy (PTT) has attracted widespread attentions because of its strong tissue penetration and favorable biosafety (Huang et al., 2006). Targeted injection of photothermal agents into tumors converts absorbed light energy into heat energy, causing structural destruction, apoptosis, or necrosis of tumor cells (Bechet et al., 2008). PTT can increase the tumor tissue temperature to approximately 47°C within 10 min, leading to the irreversible destruction of the tumor tissue by causing cell membrane damage and protein degradation (Li et al., 2014). Therefore, to ensure the effectiveness of PTT, preparing a photothermal agent with a high photothermal conversion rate and biocompatibility is essential.

Classical photothermal agents mainly include noble metal nanomaterials (e.g., germanium, palladium, and gold) (Huang et al., 2008; Li et al., 2016; Liu et al., 2016), carbon-based nanomaterials (e.g., carbon nanotubes and graphene oxide) (Samanta et al., 2008; Robinson et al., 2011; Yang et al., 2012), organic dyes (e.g., indocyanine green) (Zhou et al., 2016), and two-dimensional transition metal sulfides (Song et al., 2015). Noble metal nanoparticles, especially silver, gold and platinum, have been widely used in the biomedical field, such as the diagnosis and treatment of cancer, HIV, tuberculosis and Parkinson disease (Rai et al., 2016). In recent years, Copper chalcogenide nanomaterials, such as CuxS, CuxSe, and CuxTe (x = 1 or 2), have attracted widespread attentions as effective substitutes for gold nanomaterials (Li et al., 2010; Hessel et al., 2011; Li et al., 2013). In 2010, Li and Chen’s group first proposed that CuS NPs could induce the photothermal destruction of HeLa cells in a laser dose- and nanoparticle concentration-dependent manner under NIR irradiation at 808 nm and could exert minimal cytotoxic effects similar to those of gold nanoparticles (Li et al., 2010). Compared with gold nanomaterials, CuS NPs are cheaper, easier to synthesize, less toxic, and exhibit stable near-infrared absorption characteristics (Guo et al., 2013; Xiao, 2014). Because of the near-infrared absorption mechanism of d–d energy level transition, CuS NPs have a higher maximum absorption wavelength and absorption intensity and are generally unaffected by particle size, shape, and environmental factors (Chen et al., 2015; Zhang et al., 2015). Guo et al. (2013) synthesized PEG-HCuS NPs and PEG-HAu NPs and injected them through the tail vein. After 3 months of the intervention, neither of the two nanomaterials caused significant toxic damage to mice. However, gold NPs accumulated in the body, whereas CuS NPs were biodegraded and thus had more long-term biosafety. In addition, compared with CuS NPs, organic dyes may win out in terms of biodegradability and biocompatibility (Liu et al., 2019). But many organic dyes suffer from aggregation-induced quenching (ACQ) due to their low molecular weight and low solubility in aqueous media, thus showing poor optical stability (Weng and Liu, 2021). Generally, CuS NPs, as inorganic photothermal transduction agents, own higher photothermal conversion efficiency and better photothermal stability than organic dyes. Not only that, most organic dyes are with non-targeting or extremely low targeting property, and are rapidly cleared by the liver and kidney with short blood circulation half-life (Luo et al., 2011). In order to improve this defect, in the process of the chemical conjugation with cancer-specific ligands, the structures of organic dyes are easily damaged, thus losing their original functions. These defects limit the application of organic dyes in cancer photothermal therapy, and the development of multifunctional dyes with native or acquired cancer-targeted capacities remains an important topic. Therefore, compared with organic dyes, CuS NPs are more suitable photothermal transducers for PTT.

Due to the biocompatibility and biosafety of pure CuS NPs cannot be guaranteed, so it is necessary to combine some surface modifiers to design CuS NPs with better performance. Currently, bovine serum albumin (BSA) and polyethylene glycol (PEG) are the most commonly used surface modifiers. PEG can endow CuS NPs water-solubility and improve the biocompatibility. CuS@DSPE-PEG NPs (200 μL, 1 mg/mL) synthesized by Huang et al. (2015) were passively targeted to tumor sites through tail vein injection in mice, and they significantly reduced tumor volume under laser irradiation (Huang et al., 2015). Histological and blood biochemical analyses 24 h after injection revealed that CuS@DSPE-PEG NPs did not demonstrate marked toxicity. However, some studies have also shown that CuS@PEG NPs are only well preserved at 4°C. In both *in vitro* and *in vivo* experiments, CuS@PEG NPs degraded rapidly at 37°C within 1 week, and the generated Cu^2+^ may cause the increase of free radicals and trigger the inflammation (Shi et al., 2019). This feature reduces the biosafety of CuS@PEG NPs on the one hand, and is not conducive to photothermal ablation of tumors on the other hand. BSA has the characteristic of chelating metals, which can not only improve the biocompatibility of CuS NPs, but also help the nanoparticles to resist the scavenging mechanism of the organism and increase the biosafety. CuS-BSA NPs synthesized by Wan et al. (2019) effectively ablated HeLa cells under NIR irradiation. CuS-BSA NPs at concentrations below 45 μg/mL were not significantly toxic to HeLa cells (Wan et al., 2019). Besides, the BSA protein coat endows the synthesized CuS@BSA NPs with the ability to further functionalize. Yan et al. (2019) successfully synthesized CuS@BSA-RGD NPs by covalently grafting cyclic arginine–glycine–aspartic acid (cRGD) onto BSA *via* amidation reaction, which increased the tumor-targeting ability of the nanoparticles. And after intravenous administration to orthotopic HCC mice, CuS@BSA-RGD NPs markedly accumulated in hepatoma tissue, enabling the high-sensitivity photoacoustic visualization of tumor tissue (Yan et al., 2019). CuS@BSA-NB2 synthesized by covalently linking nanobody (NB2) to BSA had the higher targeting for breast cancer with high expression of human epidermal growth factor receptor-2 (HER2) (Ying et al., 2022). Chen et al. (2021) labeled the synthesized BSA@CuS-PEG nanocomposites with Cy5.5, a fluorescent dye, to endow the nanomaterials with optical imaging capabilities (Chen et al., 2021). Their successful labeling was attributed to the presence of the BSA coat. Therefore, we believe that BSA has higher biosafety than PEG, and BSA will be a better choice in future functional studies of CuS NPs. In addition to BSA and PEG, CuS NPs synthesized using xylan (Huang X. et al., 2017), quaternized chitosan (Huang et al., 2019), Vancomycin (Zou et al., 2020), and corn stalk (Xiong et al., 2019) as templates and stabilizers can be used for the photocatalytic treatment and sterilization of wastewater.

CuS NPs can selectively target the tumor tissue and prevent damage to healthy tissue present between the tumor and external light source, they are ideal candidates for PTT (Tian et al., 2013). At present, the synthesis methods of CuS NPs are diverse and the complexity is inconsistent, which is not conducive to clinical application. Moreover, there are few *in vivo* experimental data for biosafety evaluation, and the photothermal effect research of CuS NPs mainly focuses on *in vitro* experiments. Therefore, it is urgent to synthesize CuS NPs with low toxicity, good stability and simple synthesis method and further enrich the relevant data of tumor tissue photothermal ablation *in vivo* experiments. In this study, in view of the excellent biocompatibility and biosafety of BSA, sheet-like CuS-BSA NPs with a particle size of about 30 nm were synthesized by a facile one-pot strategy using BSA as a biological modifier and were physically characterized. The mechanism of CuS-BSA NPs-induced photothermal ablation of tumor cells was investigated by constructing a H22 hepatocellular carcinoma tumor-bearing mouse model.

## Materials and methods

### Chemicals and animals

Bovine serum albumin (BSA) was purchased from Shanghai Shisheng Sibas Advanced Technology Co., Ltd. (Shanghai, China). Copper nitrate (AR, 99.0%–102.0%), thioacetamide (AR, 99.0%), and nitric acid (AR, 65.0%–68%) were purchased from Sinopharm Chemical Reagent Co., Ltd. (Shanghai, China). Ultrapure water (18.2 MΩ) used in all experiments was prepared with Milli-Q^TM^Direct (Merck China, Shanghai, China).

Six-week-old male Kunming mice (18–22 g) were purchased from Qingdao Daren Fucheng Animal Husbandry Co., LTD. H22 hepatocellular carcinoma ascites tumor mice were purchased from the Institute of Materia Medica, Shandong Academy of Medical Sciences. All animals were maintained in a standard environment with 12 h light/dark cycle, temperature (22–24°C), and humidity (40–60%). Mice had free access to standard mouse chow and water. All the animal experiments were approved by Animal Care and Use Committee of Qingdao University and carried out under the institution guidelines for ethical animal use.

### Synthesis of CuS-BSA NPs

CuS-BSA NPs were synthesized by a facile one-pot strategy. BSA solution (5 mg/mL, 5 mL) was mixed with copper nitrate solution (10 mM, 5 mL) and stirred for 30 min at room temperature at 1,200 r/min with Magnetic stirrer (UM-4T). The pH of the mixture was adjusted to 3.0 with dilute nitric acid solution and then 0.5 mL thioacetamide solution at a concentration of 0.2 M was added. The mixture was stirred until the color of the solution changed from light blue to yellow. Then the mixed solution was quickly moved to the collector type constant temperature heating magnetic stirrer (DF-101S) for 2 h, and during which time the mixture was kept constant at 90°C. The mixture changed rapidly from pale yellow to dark green. The obtained CuS-BSA NPs solution was condensed by rotary evaporation, and then diluted to a constant volume to obtain a CuS-BSA NPs solution with a mass concentration of 300 μg/mL.

### Characterization of CuS-BSA NPs

CuS-BSA NPs was diluted with ultrapure water, mixed with ultrasound, dropped onto a copper net, and air-dried naturally. The morphological characteristics of CuS-BSA NPs were observed with a transmission electron microscope (FEI TECNAI G2 Spirit TWIN, FEI, United States).

The particle size distribution and zeta potential of the prepared CuS-BSA NPs were measured at 25°C with a Malvern Zetasizer (Zetasizer Nano ZS, Malvern, United Kingdom), and the pH value of the solution was measured with a pH test paper.

Using ultrapure water as a reference, 0.3 mL CuS-BSA NPs solution was diluted to 3 mL in ultrapure water, phosphate buffered saline (PBS) and Dulbecco’s modified eagle medium (DMEM), respectively. The absorption spectra of CuS-BSA NPs in the three solvents were measured by a spectrophotometer (UV-1800PC, Shanghai Mapada, China). The wavelength was 200–1,000 nm.

CuS-BSA NPs were directly glued to the conductive adhesive, and gold was sprayed for 45s with a sputtering coater (Oxford Quorum SC7620, United Kingdom) at a working current of 10 mA. Scanning electron microscopy (TESCAN MIRA LMS, Czech Republic) was used to capture the sample morphology and element mapping analysis.

The CuS-BSA NPs solution was diluted with PBS to obtain 120 μg/mL CuS-BSA NPs solution (3 mL), irradiated with a 980 nm laser (Hite Photoelectric, China) at a power density of 1.5 W/cm^2^ for 15 min, and then cooled to room temperature after 20 min. The absorption spectra of CuS-BSA NPs in the wavelength range of 200–1,000 nm were measured before heating and after heating and cooling.

The CuS-BSA NPs solution was lyophilized into powder by a freeze dryer (ScientZ-10N, Ningbo Xinzhi Biotechnology Co., LTD.), a small amount of which was mixed with potassium bromide and crushed thoroughly under infrared baking lamp. The mixed powder was pressed into thin slices in a mold and the infrared spectrum was measured by Fourier transform infrared spectrometer (Nicolet ECO 2000, Thermo Fisher Technologies, USA). The infrared spectra of BSA powder were measured by the same method.

### Study on photothermal effect of CuS-BSA NPs *in vitro*

The photothermal heating effect of CuS-BSA NPs solution with different concentrations was measured. The CuS-BSA NPs were configured with ultrapure water to a series of concentration gradients of 7.5, 15, 30, 60, 120, 240, and 300 μg/mL, and irradiated at 980 nm with a power density of 1.5 W/cm^2^ for 10 min at 25°C. The temperatures were recorded every 10 s during irradiation.

The photothermal heating effect of CuS-BSA NPs solution (120 μg/mL) under different power densities that were 0.5, 1, 1.5, 2.0, and 3.0 W/cm^2^ for 10 min was measured. The temperatures were recorded every 10 s during irradiation.

The photothermal stability of the CuS-BSA NPs solution was measured. CuS-BSA NPs solution (120 μg/mL, 3 mL) was irradiated by a 980 nm laser with a power density of 1.5 W/cm^2^ for 15 min and then cooled to room temperature. Another identical sample was irradiated for 5 min under the same conditions, then the laser closed for 5 min, and the cycle was repeated 5 times. The temperatures were recorded every 10 s for both treatments.

### Toxicity of CuS-BSA NPs *in vivo*

After 1 week of adaptive feeding, 36 Kunming mice were randomly assigned into 6 groups according to their body weight, with 6 mice in each group. The concentration of CuS-BSA NPs diluted in PBS was 300 μg/mL. Different one-time doses of CuS-BSA NPs solution were injected through the tail vein. The doses were 1800, 3,600, 5,400, 7,200 and 9,000 μg/kg. Measured body weight every other day. The control group was injected with PBS solution at a dose of 6 mL/kg by tail vein. After 14 days, the mice were sacrificed, and the liver, spleen, kidney and thymus were taken and weighed, and the thymus index and spleen index were calculated. The liver, spleen and kidney were fixed with 10% neutral formalin for pathological observation.

### Establishment of H22 hepatoma-bearing mouse model

48 Kunming mice were fed adaptively for 7 days prior to experimental enrollment. After abdominal disinfection of H22 hepatoma ascites tumor mice, 5 mL ascites were extracted in a sterile environment and diluted with PBS, and the cell number was adjusted to 2×10^6^/mL. 0.2 mL of H22 cell suspension was inoculated subcutaneously on the back of the right hind limb of each male Kunming mouse.

### CuS-BSA NPs mediated photothermal ablation of tumors

Five days after inoculation with H22 cell suspension, the tumor formation rate was 100%. According to tumor volume, 48 mice were randomly assigned into six groups with 8 mice in each group. At the end of the experiment, the number of mice with complete date was 6 per group. Specific groups were as follows: blank control group (PBS_T_ group): intratumoral injection of PBS solution; single irradiation group (PBS_T_ + NIR group): intratumoral injection of PBS with 980 nm near-infrared light irradiation; CuS_T_ group: CuS-BSA NPs solution was injected intratumorally; CuS_T_ + NIR group: CuS-BSA NPs solution was injected intratumorally and irradiated with 980 nm near-infrared light; CuS_V_ group: CuS-BSA NPs solution was injected into the tail vein of mice; CuS_V_ + NIR group: CuS-BSA NPs solution was injected into the tail vein of mice and irradiated with 980 nm near-infrared light. The concentration of CuS-BSA NPs diluted in PBS was 100 μg/mL, and the dose was 600 μg/kg. PBS solution was injected at a dose of 6 mL/kg. After anesthesia, the subcutaneous tumors were irradiated with the near-infrared light with a power density of 2.0 W/cm^2^ and wavelength of 980 nm for 10 min, which was performed once a week for a total of 3 times. During the experiment, the body weight and tumor volume of the mice were measured every 2 days. Three weeks later, the liver, spleen, kidney, and thymus were harvested and the tumor was completely excised and weighed. Some parameters were calculated according to the formula described below:

Tumor volume=(a×b2)/2

$$Tumor\;weight/body\;weight\;\left(T/B\right)\;ratio=W_{Tumor}/W_{\mathrm{Body}}\times 100\%$$

$$Tumor\;growth\;inhibition\;rate\;\left(\%\right)=\left(W_{\mathrm{Control}}-W_{\mathrm{Treated}}\right)/W_{\mathrm{Control}}\times 100\%$$

(a: length of tumor; b: width of tumor; W _Control_: mean tumor weight of mice in the PBS_T_ group; W _Treated_: mean tumor weight of mice in other treated group; W _Tumor_: mean tumor weight; W _Body_: mean body weight).

Thymus index=thymus weight (mg)/body weight (g)

Spleen index=spleen weight (mg)/body weight (g)

### Pathological section

Samples of liver, spleen, kidney, and tumor tissue were fixed with 10% neutral buffered formaldehyde. Thin slices (5 μm) from each sample were stained with hematoxylin and eosin (H&E) and photographed under an optical microscope.

### Immunohistochemistry

Immunohistochemistry was used to verify the expression of Cleaved caspase-3 and Cleaved caspase-9 of tumor tissue. The tumor tissue sections were incubated overnight at 4°C with Cleaved Caspase-3 (1:1,000 dilution) (Cell Signaling Technology Cat# 9664, RRID:AB_2070042) or Cleaved Caspase-9 (1:1,000 dilution) (Abcam Cat# ab52298, RRID:AB_868689) primary antibodies, washed three times with PBS and then were incubated with HRP-labeled secondary goat anti-rabbit antibody (1:1,000 dilution) (Abcam Cat# ab6721) for 30 min at room temperature. After washing in PBS for three times, all sections were incubated with freshly prepared DAB solution for 15 min at room temperature and counterstained with hematoxylin. Pictures were taken with a microscope (Changfang, Shanghai, China), and quantified with ImageJ software (NIH, United States).

### Statistical analysis

SPSS 26.0 software was used for statistical analysis. Data results were expressed as mean ± standard deviation (SD). In the *in vivo* toxicity experiment, for normally distributed data, one-way ANOVA was used to statistically analyze the differences between multiple groups. In the *in vivo* photothermal effect experiments, the interaction between CuS-BSA NPs and NIR was first tested, and two-way ANOVA was used to assess differences when a significant interaction was detected. If the effects of CuS-BSA NPs and NIR were independent, normality and homogeneity were checked out, and one-way ANOVA was used to assess differences. Shapiro-Wilk test and Leven test were used to assess the normality and homogeneity of the data, respectively. Results were considered statistically significant when *p* < 0.05.

## Results

### Characterization of CuS-BSA NPs

**FIGURE 1:**
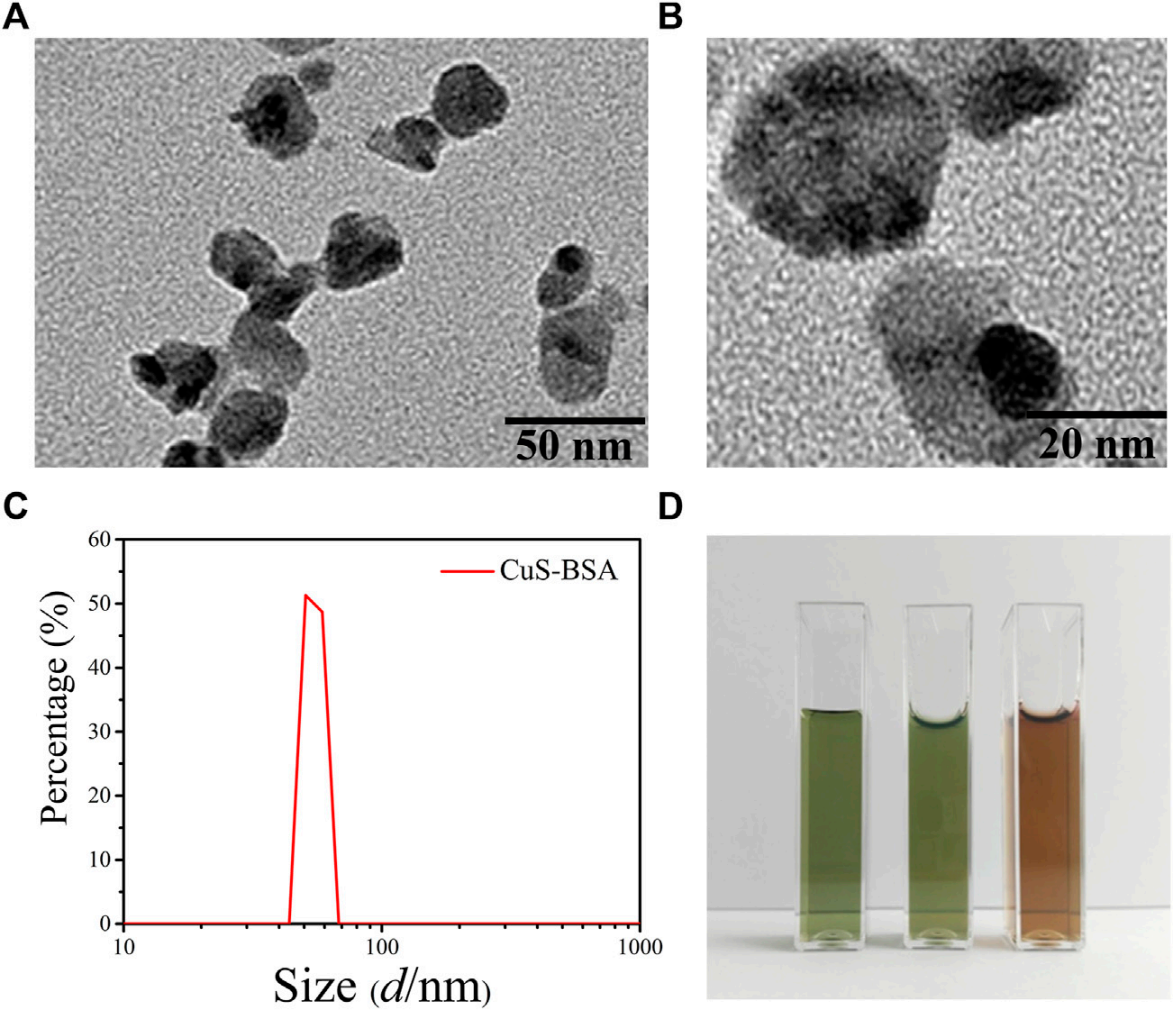
The characterization of CuS-BSA NPs. **(A)**and **(B)** The TEM images of CuS-BSA NPs. Scale bars are 50 nm and 20 nm, respectively. **(C)** DLS analysis. **(D)** Digital photos of dispersion solution of CuS-BSA NPs in different solvents. From left to right, the solvents were ultrapure water, PBS, and DMEM medium.

Under the transmission electron microscope, the morphology of the synthesized CuS-BSA NPs was a nearly circular lamellar structure with a particle size of about 30 nm, which was relatively uniform with good dispersion in the field of vision (Figures 1A,B). The particle size was similar to that of CuS-BSA NPs synthesized through different methods by Wang et al. (2015), which could be used for biomedical applications (Wang et al., 2015).

The Zeta potential of CuS-BSA NPs measured at 25°C and a pH of seven was -29.7 ± 2.31 mV. The fluid dynamic diameter was 50.7 nm. The particle size curve was unimodal and the distribution range of the peak was narrow (Figure 1C). No significant spectra changes were observed for the CuS-BSA NPs dispersed in the ultrapure water for 15 days (Supplementary Figure S1). The CuS-BSA NPs solution dispersed in three different solvents of ultrapure water, PBS, and DMEN medium was homogeneous and stable after 1 month, and there was no precipitation and flocs, indicating that the CuS-BSA NPs possessed excellent dispersibility and stability, which was suitable for the liquid environment required for cell and animal experiments (Figure 1D).

**FIGURE 2:**
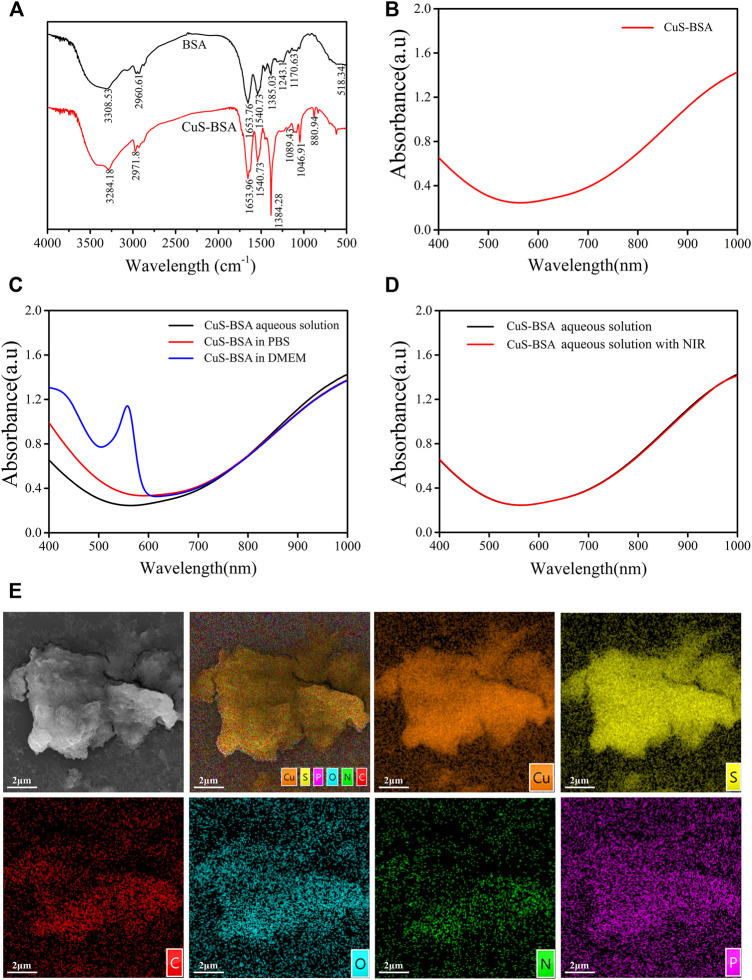
FT-IR and UV absorption spectra of CuS-BSA NPs. **(A)** The FT-IR spectra of BSA and CuS-BSA NPs. **(B)** UV absorption spectra of CuS-BSA NPs in ultrapure water. **(C)** UV absorption spectra of CuS-BSA NPs in three different solvents: ultrapure water, PBS, and DMEM medium. **(D)** UV absorption spectra of CuS-BSA NPs in PBS before and after irradiation with 980 nm near-infrared light. **(E)** SEM morphology image and element mapping analysis of CuS-BSA NPs.

The FT-IR spectra of CuS-BSA NPs and BSA (Figure 2A) revealed that the two substances had different vibration frequencies and intensities mainly in the region of 3,300–3,000 cm^−1^. The stretching vibration peak of the -NH bond in BAS changed from 3,308.53 cm^−1^ to 3,284.18 cm^−1^, and the peak of the -CH bond changed from 2960.61 cm^−1^ to 2971.8 cm^−1^, indicating that BSA formed a coordination complex with Cu^2+^. There was no obvious change in the position of the peaks between 1700 cm^−1^ and 1,100 cm^−1^. Overall, CuS-BSA NPs still retained the characteristic absorption peaks of BSA. The distributions of copper and sulfur elements and the corresponding elements contained in the BSA protein were shown in Figure 2E, which further confirmed that BSA was indeed bound to CuS. Full-wavelength absorption spectrum (Figure 2B) showed that the CuS-BSA NPs aqueous solution had a strong absorption peak at 980 nm, which further proved that CuS-BSA NPs had a significant absorption characteristic of near-infrared light. In addition, there was a strong absorption peak of BSA at about 280 nm (not shown). In the three different solvents of ultrapure water, PBS, and DMEM medium, there was no significant difference in the full-wavelength absorption spectra of CuS-BSA NPs (Figure 2C). Our results also showed that its absorption spectra did not change significantly before and after 980 nm NIR irradiation (Figure 2D). To sum up, CuS-BSA NPs possessed good biological applicability and photothermal stability.

### Photothermal effect of CuS-BSA NPs

**FIGURE 3:**
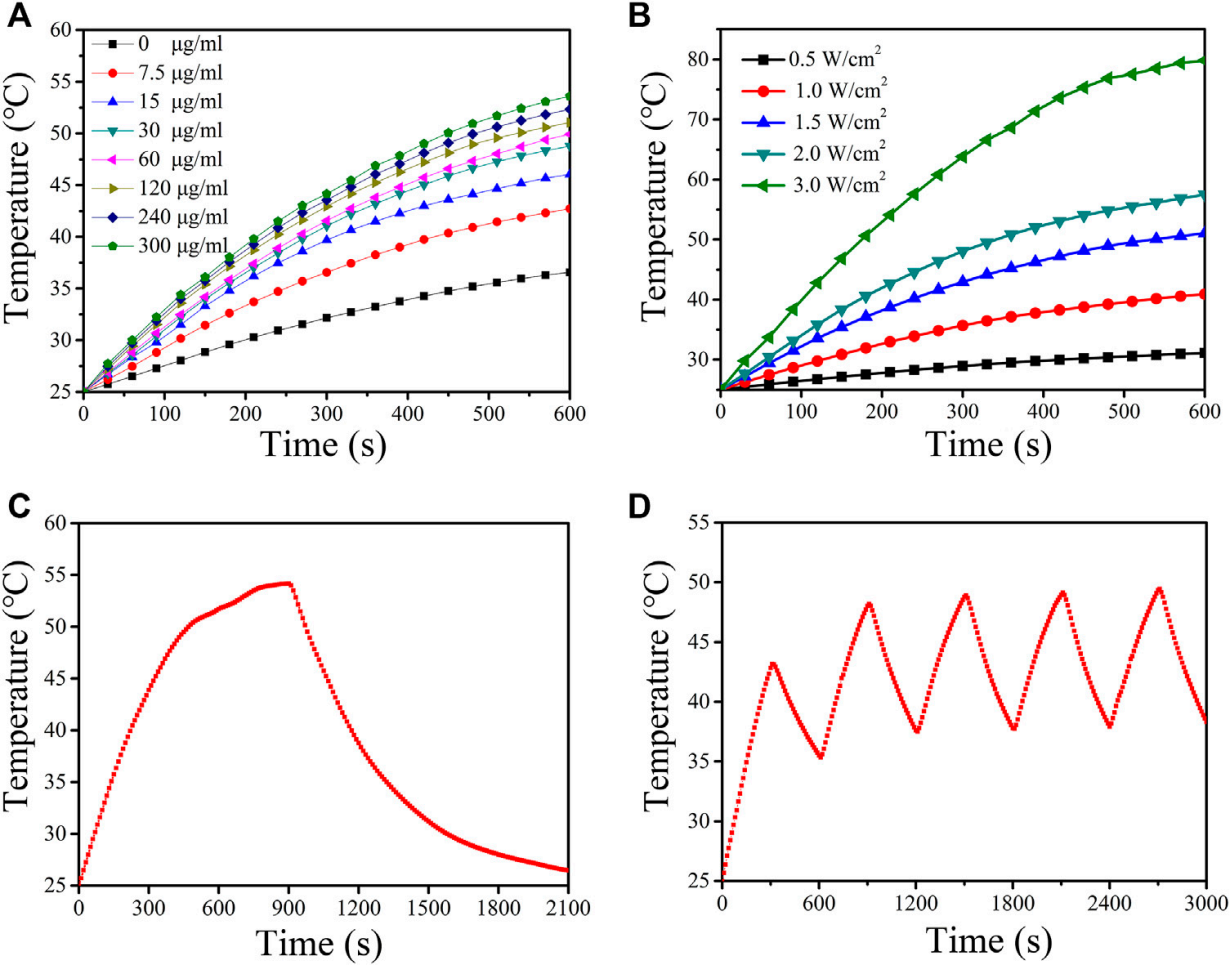
Photothermal effect of CuS-BSA NPs. **(A)** Photothermal conversion of CuS-BSA NPs aqueous solution with different concentrations *in vitro* under irradiation of a 980 nm laser (1.5 W/cm^2^) for 10 min. **(B)** Photothermal conversion of CuS-BSA NPs aqueous solution (120 μg/mL) with a 980 nm laser at different power densities for 10 min. **(C)** Temperature curve of CuS-BSA NPs aqueous solution (120 μg/mL) during irradiating with 980 nm laser (1.5 W/cm^2^) and cooling process. **(D)** The heat-cool loop of CuS-BSA NPs aqueous solution (120 μg/mL) *in vitro* under irradiation of a 980 nm laser (1.5 W/cm^2^) for five heat-cool cycles.

To evaluate the photothermal properties of CuS-BSA NPs, the temperature rise test was carried out. Irradiated with a 980 nm laser (1.5 W/cm^2^) for 10 min, the temperature of CuS-BSA NPs aqueous solution with different concentrations all increased, and the photothermal effect became more and more obvious with the increase of concentration (Figure 3A). When the concentration was up to 300 μg/mL, the temperature rose by 28°C, while the ultrapure water as a blank control only rose by 11°C. After irradiation with a 980 nm laser at different power densities for 10 min, when the power density was 3.0 W/cm^2^, the temperature increased by 45°C, and the temperature increased by only 5°C when the power density was the lowest 0.5 W/cm^2^ (Figure 3B). These results indicated that the photothermal effect of CuS-BSA NPs was concentration-dependent and light-intensity-dependent. After a 980 nm laser (1.5 W/cm^2^) irradiated the CuS-BSA NPs aqueous solution (120 μg/mL) for 15 min, the temperature reached 55°C, and it could be lowered to room temperature after 20 min of light cooling (Figure 3C). After the heat-cool loop of CuS-BSA NPs aqueous solution for five cycles, the last four peaks had no obvious change (Figure 3D). In conclusion, the CuS-BSA NPs synthesized in this study had good photothermal stability.

### Toxicity of CuS-BSA NPs *in vivo*

**FIGURE 4:**
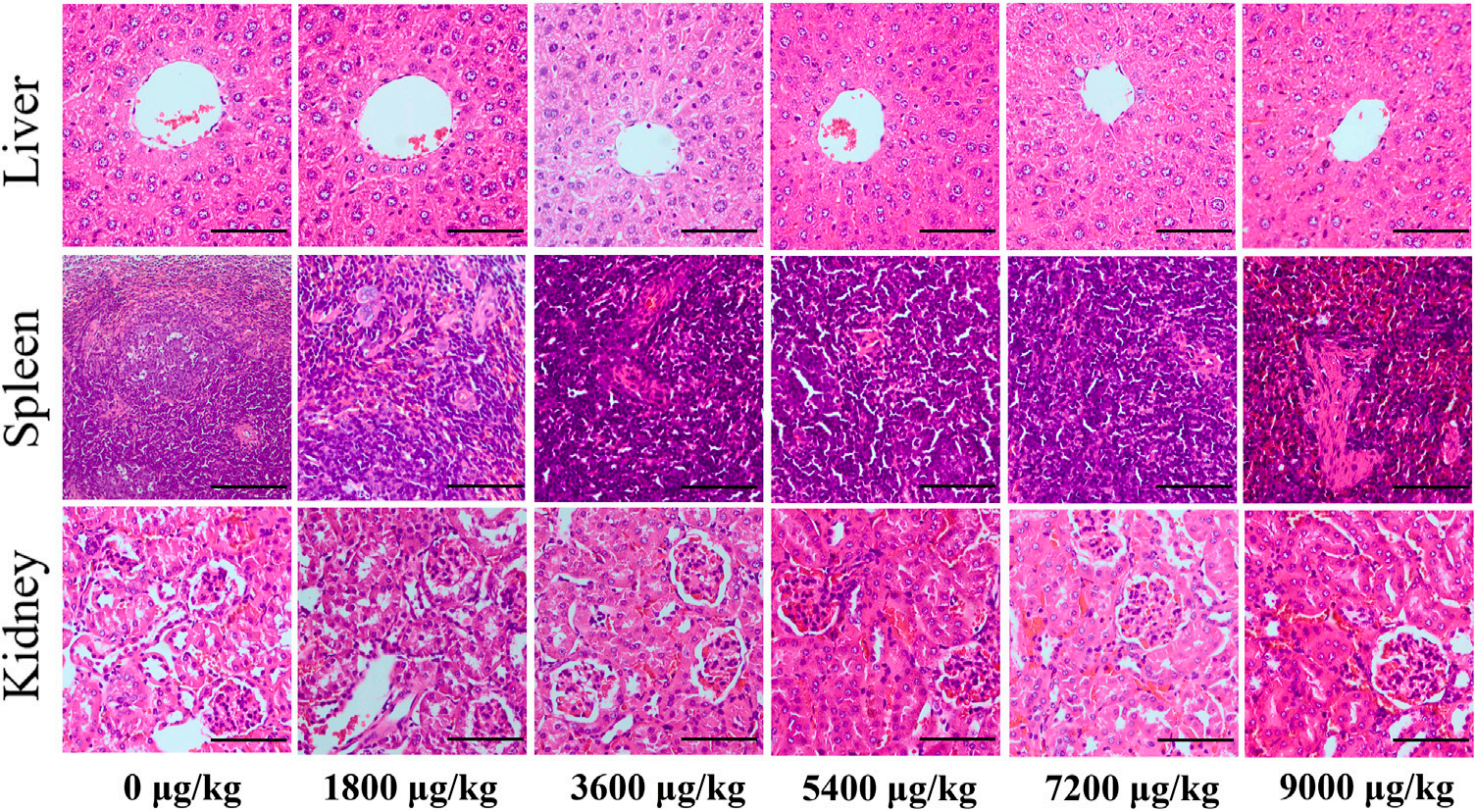
Pathological sections of the liver, spleen, and kidney from mice in the *in vivo toxicity* evaluation of CuS-BSA NPs (×400). Scale bars represent 100 μm.

There was no significant difference in thymus index and spleen index among groups after tail vein injection of different doses of CuS-BSA NPs (*p* > 0.05) (Supplementary Table S1). The pathological results (Figure 4) showed that when the CuS-BSA NPs were in the range of 1800–7,200 μg/kg, there was no obvious damage to the structures of the liver, kidney and spleen. But when the concentration of nano-copper sulfide reached 9,000 μg/kg, a small piece of necrotic area could be found in the spleen pathological section. Toxicity evaluation provided a reference safe concentration range for CuS-BSA NPs for biomedical applications.

### Photothermal therapy *in vivo*

**FIGURE 5:**
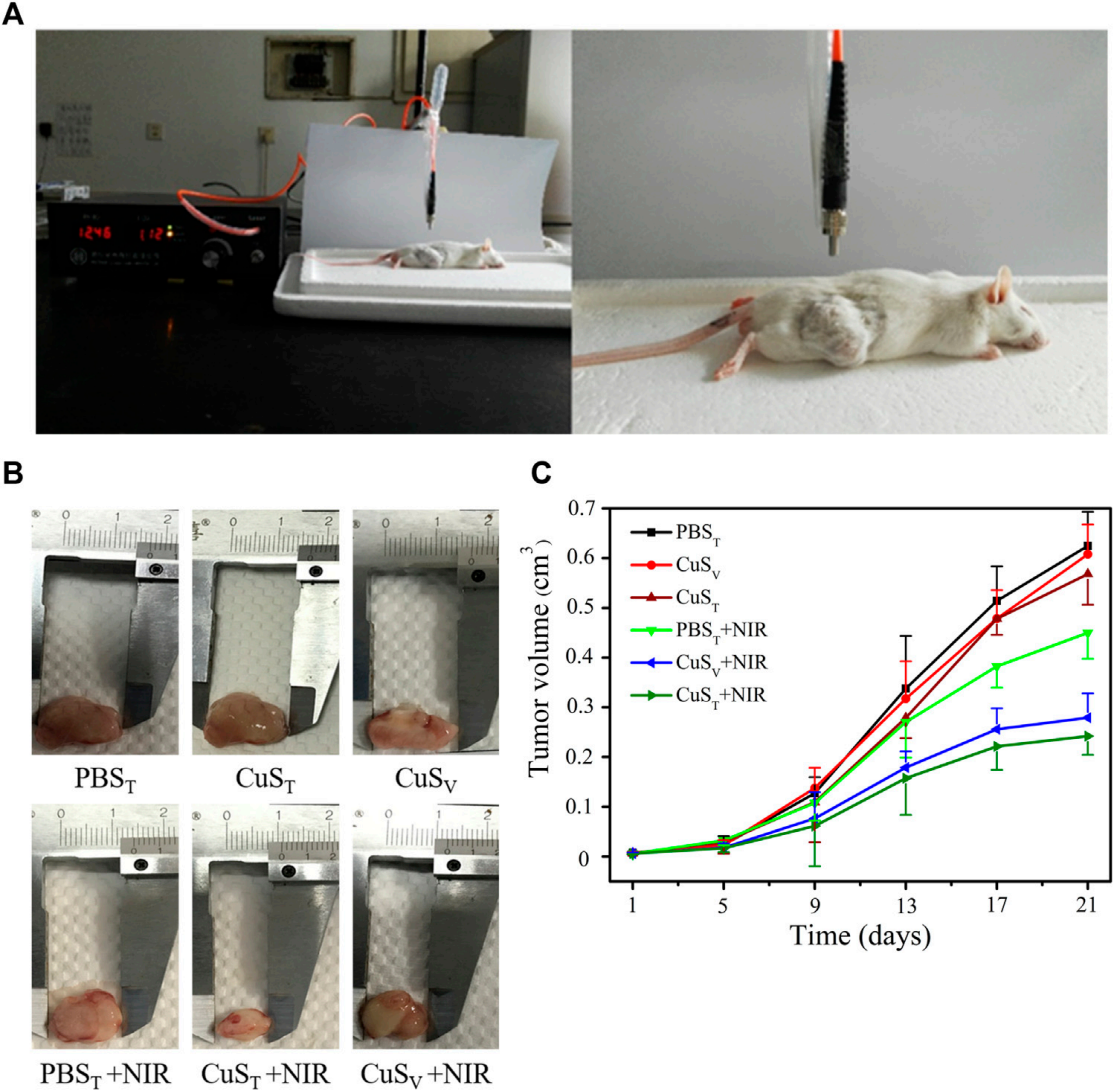
Effects of photothermal therapy on tumor growth in H22 hepatoma-bearing mice. H22 cells were injected subcutaneously into the back of the right hind limb of male Kunming mice aged 6 weeks. Mice were randomly divided into six groups after tumor formation according to tumor volume. PBS_T_ group, intratumoral injection of PBS solution; PBS_T_ + NIR group, intratumoral injection of PBS solution and irradiation with 980 nm near-infrared light (2.0 W/cm^2^); CuS_T_ group, intratumoral injection of CuS-BSA NPs solution; CuS_T_ + NIR group, intratumoral injection of CuS-BSA NPs solution and irradiation with 980 nm near-infrared light (2.0 W/cm^2^); CuS_V_ group, caudal vein injection of CuS-BSA NPs solution; CuS_V_ + NIR group, intratumoral injection of CuS-BSA NPs solution and irradiation with 980 nm near-infrared light (2.0 W/cm^2^). The concentration of CuS-BSA NPs diluted in PBS was 100 μg/mL, and the dose was 600 μg/kg. PBS solution was injected at a dose of 6 mL/kg. Photothermal treatment was performed once a week for a total of 3. **(A)** Photothermal treatment of H22 tumor-bearing mice with a near-infrared heating device. **(B)** Representative images of tumor removal in different groups of mice at the end of treatment. **(C)** The tumor volume at the beginning of photothermal therapy was taken as the initial volume, and the difference between the tumor volume at each measurement and the initial tumor volume was taken as the *Y*-axis, and time was *X*-axis to draw the tumor volume growth curve (N = 6 per group).

**FIGURE 6:**
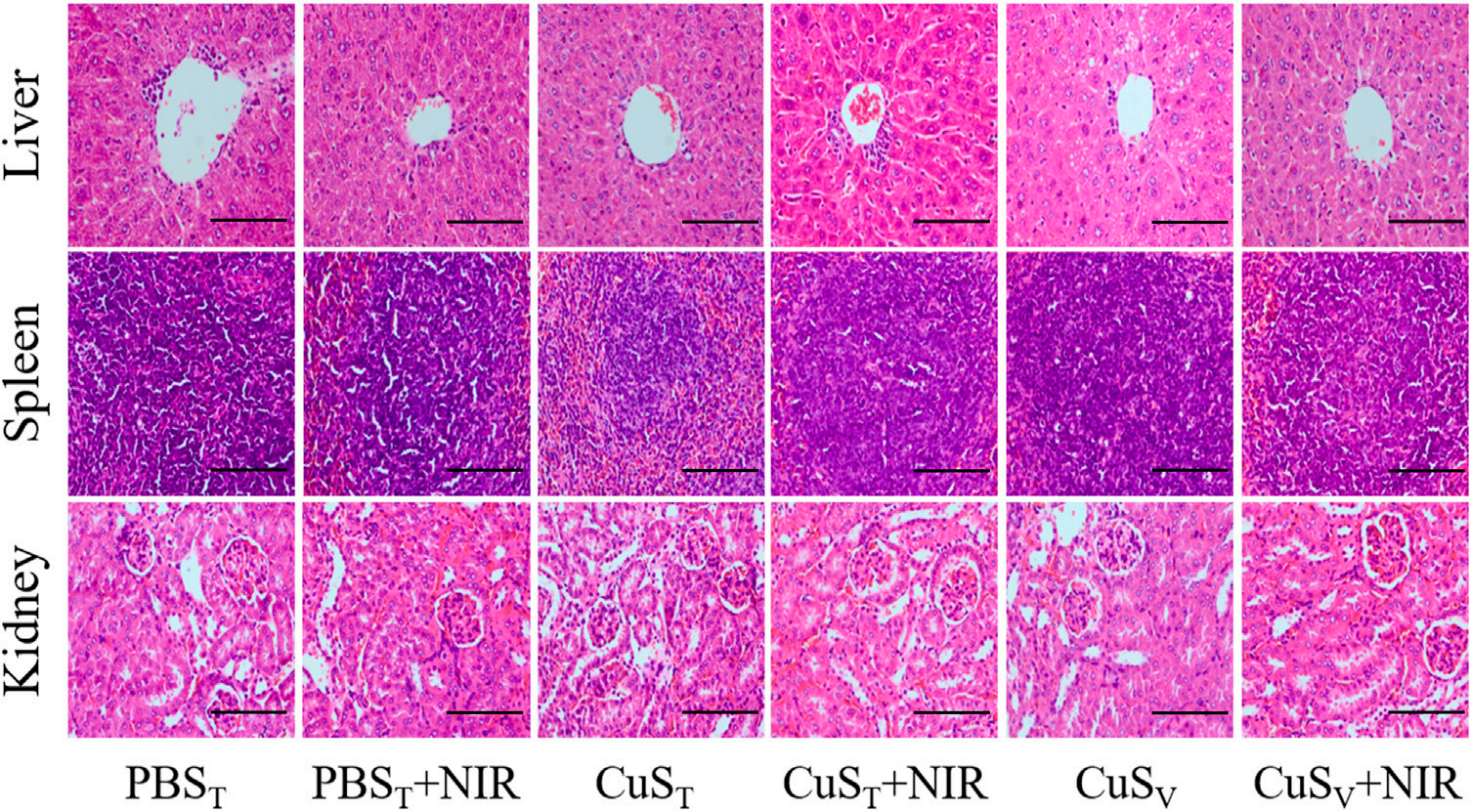
Pathological sections of the liver, spleen, and kidney in the photothermal experiment on H22 hepatoma-bearing mice (×400). Scale bars represent 100 μm.

To further examine the photothermal conversion effect of CuS-BSA NPs on tumor killing *in vivo*, we opted for a lower dose of 600 μg/kg and performed photothermal therapy on H22 tumor-bearing mice using a near-infrared heating device as shown in Figure 5A. When CuS-BSA NPs were injected intratumorally, the syringe needle would cause invasive damage to the tumor and thus affect tumor growth. Therefore, we also adopted the method of caudal vein injection. The tumor volume in the PBS_T_ group, CuS_T_ group, CuS_V_ group, and PBS_T_ + NIR group increased significantly, while the tumor grew very slowly in the CuS_T_ + NIR group and CuS_V_ + NIR group (Figures 5B,C). Compared with the PBS_T_ group, there was no significant difference in tumor volume between the CuS_T_ group and CuS_V_ group. The inhibition of tumor growth was mainly due to the photothermal effect of CuS-BSA NPs. Furthermore, the amount of CuS-BSA NPs transported through the bloodstream to the tumor site during caudal vein injection was less than that in the tumor site during direct intratumoral injection. However, no remarkable difference in tumor volume was found between the CuS_T_ group and CuS_V_ group, nor between the CuS_T_ + NIR group and CuS_V_ + NIR group. The tumor mass ratio of the CuS_T_ + NIR group and CuS_V_ + NIR group was significantly smaller than that of the PBS_T_ + NIR group (*p* < 0.05), and the tumor inhibition rates were 48.8% and 41.5%, respectively (Table 1), which demonstrated that the overall effects of intratumoral injection and caudal vein injection of CuS-BSA NPs were similar. The tumor volume of the PBS_T_ + NIR group was slightly smaller than that of the PBS_T_ group, which proved that NIR irradiation alone could also inhibit tumor growth to a certain extent, but the effect would be significantly enhanced in the presence of CuS-BSA NPs. In addition, the organ index of the spleen and thymus (Supplementary Table S2) and the hematoxylin and eosin (H&E) stained sections of the liver, spleen and kidney (Figure 6) showed that no obvious signs of organ damage were observed.

**TABLE 1:** Comparison of tumor weight, tumor weight/body weight ratio, and tumor growth inhibition rate in each group (N = 6 per group).

	Tumor weight(g)	Bodyweight(g)	T/B ratio (%)	Tumor growth inhibition rate (%)
Group				
PBS_T_	1.23 ± 0.32	35.6 ± 3.65	3.45 ± 0.67	—
PBS_T_ + NIR	1.08 ± 0.27	36.7 ± 4.72	2.94 ± 0.73	12.20
CuS_T_	1.12 ± 0.13	34.3 ± 4.83	3.26 ± 0.58	8.90
CuS_T_ + NIR	0.63 ± 0.09^a^	37.6 ± 5.50	1.68 ± 0.34^a^	48.80
CuS_V_	1.18 ± 0.43	33.8 ± 3.78	3.78 ± 0.63	4.10
CuS_V_ + NIR	0.72 ± 0.24^a^	36.2 ± 5.98	1.98 ± 0.61^a^	41.50

Data are presented as the mean ± SD.

^a^ Compared with PBS_T_ + NIR, group, *p* < 0.05.

**FIGURE 7:**
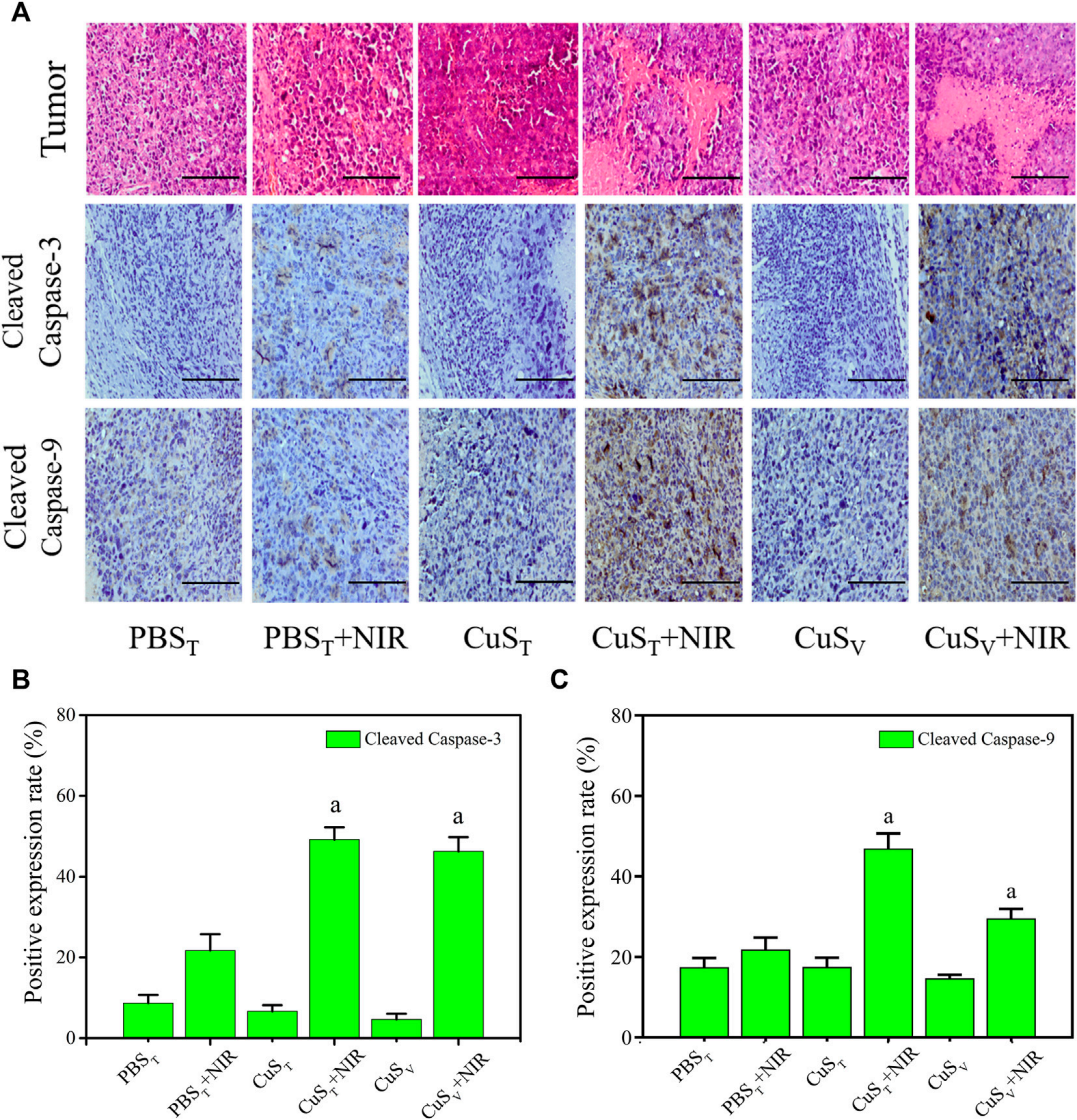
Apoptosis level of tumor tissue cells after photothermal therapy. **(A)** Pathological section of tumors with hematoxylin-eosin staining (the first line) and immunohistochemistry section (the rest lines). Scale bars represent 100 μm. **(B)** and **(C)** Quantitative analysis of immunohistochemistry (N = 3 per group). a: compared with the PBS_T_ + NIR group, *p* < 0.05.

According to histopathological observation (Figure 7A), the tumor cells in each group had typical morphological characteristics of the tumor, including polygonal, different sizes, and abundant cytoplasm. Tumor sections in the CuS_T_ + NIR group and CuS_V_ + NIR group had large necrotic areas, the cell structure disappeared, and the nuclei were fragmented and dissolved. The photothermal effect of CuS-BSA NPs led to cell death and tumor growth inhibition. Scattered areas of necrosis also appeared in the PBS_T_ + NIR group, which was less severe than in the first two groups. The tumor tissue of the other three groups did not show such obvious necrosis foci.

Apoptosis and necrosis are not two forms of cell death in isolation. Certain factors that cause apoptosis also can cause necrosis, and apoptosis and necrosis can be transformed into each other (He et al., 2009; Zhang et al., 2009). We examined the active spliced forms of apoptosis initiator caspase-9 and executor caspase-3. Our immunohistochemical results (Figure 7A) showed that cleaved caspase-9 and cleaved caspase-3 protein expressions were different degrees of cytoplasmic hyperchromic in the PBS_T_ + NIR group, CuS_T_ + NIR group, and CuS_V_ + NIR group. In the CuS_T_ + NIR group and CuS_V_ + NIR group, cleaved caspase-9 and cleaved caspase-3 protein expression levels were significantly higher than those in the PBS_T_ + NIR group (*p* < 0.05) (Figures 7B,C). The above results showed that the photothermal conversion effect of CuS-BSA NPs could induce the apoptosis of the tumor cells, thereby inhibiting the growth of tumors.

## Discussion

In this study, flake CuS-BSA NPs with a particle size of 30 nm were facilely synthesized, with good stability and biocompatibility. In the evaluation of *in vivo* toxicity, CuS-BSA NPs did not cause significant pathological changes in liver, spleen and kidney of mice in a wide concentration range of 1800–7,200 μg/kg. The photothermal effect of CuS-BSA NPs, excited by near-infrared light at 980 nm, could cause coagulative necrosis and apoptosis of tumor tissue. In general, CuS-BSA NPs have broad application prospects in the field of photothermal therapy of HCC.

In recent years, the development of CuS NPs has improved the biomedical and clinical applications of nanomaterials. Various methods for synthesizing CuS NPs have been developed. However, the biomedical application of CuS NPs is still in the infancy, and its biotoxicity still needs to be determined thoroughly (Goel et al., 2014; Feng et al., 2015). Nanoparticles with suitable particle size can induce enhanced permeability and retention (EPR) effect (Xu et al., 2018; Luan et al., 2021). It is generally believed that nanoparticles with a size of 10–100 nm are desirable because they can escape from renal filtration elimination and accumulate in tumors after prolonged circulation, which is beneficial for tumor photothermal therapy (Rao, 2008). Nanoparticles with too small particle size (<10 nm) are easily eliminated by kidney filtration, which is not conducive to accumulation in tumors, while nanoparticles with too large particle size are easy to accumulate non-specifically in the reticuloendothelial system (RES) organs (liver, spleen, etc.), thereby causing acute or chronic damage to the body (Choi et al., 2007; Chen et al., 2021). Therefore, the particle size largely affects the efficiency and biosafety of photothermal therapy of nanoparticles. Gold nanocages with a size of 30 nm had higher blood retention and tumor uptake levels, as well as lower liver and spleen uptake, compared to 55 nm gold nanocages (Wang et al., 2012). In addition, based on the EPR effect, drug-loaded polymeric micelles (with different diameters of 30, 50, 70 and 100 nm) could effectively accumulate in high-permeability tumor tissue, but only 30 nm micelles could penetrate poorly permeable pancreatic tumors to achieve an antitumor effect (Cabral et al., 2011). These results prompt that the particle size of 30 nm can provide the nanoparticles with better biocompatibility and biosafety than other particle sizes. The nanoparticles synthesized in our study had an average particle size of 30 nm under electron microscope, and a hydrodynamic particle size of about 50 nm. In the previous *in vitro* experiments, the cell viability was still above 80% when cultured with HeLa cells and human skin fibroblasts (HSF) at a dose of 48 μg/mL of CuS-BSA NPs for 72 h (not shown). In the *in vivo* toxicity test, when the doses of CuS-BSA NPs were in the range of 1800-7,200 μg/kg, no obvious pathological damage was observed in the liver, kidney and spleen, and the thymus index and spleen index did not change significantly, indicating that CuS-BSA NPs had low toxicity. In the photothermal ablation experiment of tumor tissue, the tail vein injection group and the intratumor injection group showed similar tumor ablation effects under the near-infrared light irradiation, indicating that CuS-BSA NPs accumulated in tumor tissue through blood circulation and EPR effect. In addition, the nanoparticle aqueous solution with too large particle size has poor stability and is prone to agglomeration and precipitation, thereby losing its proper function. For example, CuS-MoS2-SH-PEG with a particle size of 115 nm began to precipitate after 15 days, while CuS-MoS2 nanoparticles without PEG-SH modification had completely precipitated by the 15th day (Zhang et al., 2019). The CuS-BSA NPs synthesized in this study had good dispersibility and stability, and could remain stable for at least 30 days.

Thermal ablation in the tissue is dependent on the temperature increase caused by the photothermal agent under a light source and the duration of treatment required to kill tumor cells. PTT with temperatures higher than 55°C can be more effective than low-level PTT with temperatures ranging from 45°C to 55°C (Habash et al., 2007). CuS-BSA NPs synthesized in our study exerted a photothermal effect under 980 nm NIR, which exhibited concentration dependence and power density dependence. When the power density was 1.5 W/cm^2^, the photothermal heating effect was dependent on the concentration of CuS-BSA NPs. When the maximum concentration was 300 μg/mL, the temperature increased by 31°C after 10 min of irradiation. When the concentration was 120 μg/mL and the power density was 3.0 W/cm^2^, the temperature increased by 45°C. When the temperature exceeds 50°C, the cells undergo coagulation necrosis after 2 min. In the *in vitro* heating experiment, the temperature of the aqueous solution containing 120 μg/mL CuS-BSA NPs reached 55°C after irradiation at a power of 1.5 W/cm^2^ for 15 min. Even at the lowest concentration of 7.5 μg/mL, the temperature reached approximately 42°C after 10 min of irradiation. In addition, the CuS-BSA NPs used in the current study is synthesized with methods provided by Dapeng Yang, and they have reported a photothermal conversion efficiency of 24.68% on the identical material (Huang J. et al., 2017). So, it can be used as a reference for the photothermal conversion efficiency of nanoparticles in our study. These results indicated that CuS-BSA NPs demonstrated an ideal photothermal conversion efficiency.

Apoptosis is regulated by multiple genes. The caspase family comprises cysteine proteases, which are proteolytic enzymes used to specifically cleave protease and then trigger apoptosis. In both exogenous and endogenous apoptosis pathways, the activation of caspase-3 and other caspase family proteins, such as caspase-9 and caspase-7, is required to complete the apoptosis process (Zhou et al., 2000). Among studies on PTT, few have focused on the photothermal ablation mechanism of CuS NPs as photothermal agents. In this study, H22 tumor–bearing mice were used to explore the applicability of CuS-BSA NPs in PTT and to preliminarily investigate the expression of apoptotic proteins in tumor cells. Under the near-infrared laser irradiation, both in the tail vein injection group and the intratumor injection group, a large area of necrosis in the tumor tissue, as well as the up-regulation of apoptotic proteins including cleaved caspase-3 and cleaved caspase-9 were observed; these findings significantly differed from those of the blank control group, indicating that CuS-BSA NPs could induce liver cancer cells apoptosis to inhibit tumor growth during the treatment of photothermal therapy, in addition to causing coagulation necrosis of liver cancer cells through high temperature.

In summary, we used an environmentally friendly, mild, economical, and efficient method to synthesize low-toxicity CuS-BSA NPs, which could effectively inhibit tumor growth by photothermal therapy. CuS-BSA NPs are expected to be used as a photothermal agent for photothermal therapy of cancer. The limitation of our study was only using one dose and one power density in our tumor photothermal ablation experiment. Other doses and power density should be explored in future.

## Conclusion

In this study, the sheet-like CuS-BSA NPs with a particle size of 30 nm were synthesized using BSA as a modifier. CuS-BSA NPs had good dispersion and a good photothermal effect. In the evaluation of *in vivo* toxicity, when the concentration of CuS-BSA NPs was in the range of 1800–7,200 μg/kg, the mice grew well, and no pathological changes were observed in the liver, spleen and kidney. As a photothermal agent, CuS-BSA NPs could effectively inhibit tumor growth in H22 tumor-bearing mice under 980 nm NIR. CuS-BSA NPs-mediated photothermal therapy could cause coagulative necrosis and up-regulate the expression of apoptosis proteins including cleaved caspase-3 and cleaved caspase-9, which inhibited tumor growth by inducing apoptosis to achieve photothermal therapy.

## Data Availability

The materials described in the article, including all relevant raw data, will be freely available to any scientist wishing to use them for non-commercial purposes, and the data used in the current study can be obtained from the corresponding authors on a reasonable request.
