# Supplementary material for: Photothermal effects of CuS-BSA nanoparticles on H22 hepatoma-bearing mice
Source: Front Pharmacol. 2022 Oct 12;13:1029986. doi: 10.3389/fphar.2022.1029986 (PMC9596806; doi:10.3389/fphar.2022.1029986)
Supplement: Supplementary file 1 [file Table1.DOCX]

Supplementary Material


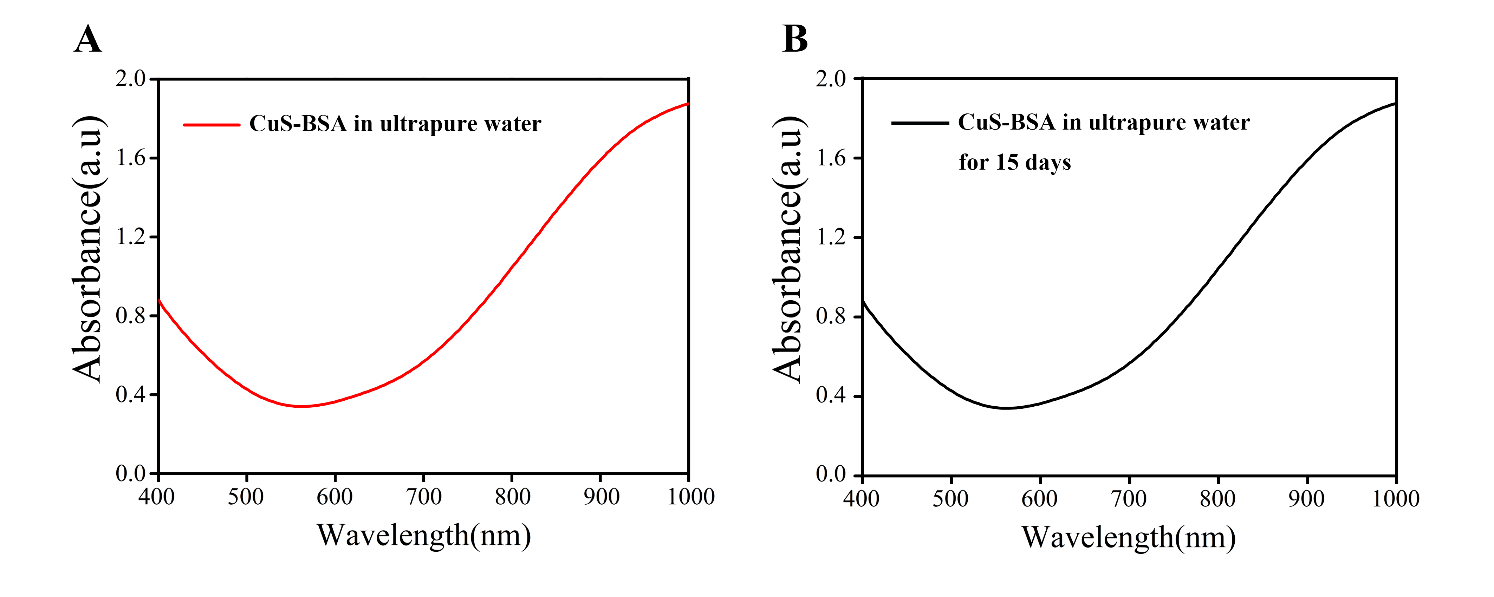


**Figure S1.** Absorption spectra of CuS-BSA NPs dispersed in ultrapure water immediately (A) and for 15 days (B).

**Table 1 Comparison of thymus index and spleen index in each group (N=6 per group)**

| Project  Group | Spleen index (mg/g) | Thymus index (mg/g) |
| --- | --- | --- |
| 0μg/kg | 6.73±1.61 | 2.12±0.81 |
| 1800μg/kg | 6.93±1.72 | 2.01±0.70 |
| 3600μg/kg | 6.68±1.97 | 1.98±1.12 |
| 5400μg/kg | 7.12±1.33 | 2.36±1.03 |
| 7200μg/kg | 6.92±1.84 | 2.56±0.5 |
| 9000μg/kg | 7.28±1.73 | 2.23±0.81 |

Data are presented as the mean ± SD.

**Table 2 Comparison of thymus index and spleen index in each group (N=6 per group)**

| Project  Group | Spleen index (mg/g) | Thymus index (mg/g) |
| --- | --- | --- |
| PBS_T_ | 5.43±1.32 | 1.89±0.65 |
| PBS_T_+NIR | 5.28±0.87 | 1.78±0.72 |
| CuS_T_ | 6.42±0.73 | 2.06±0.83 |
| CuS_T_+NIR | 5.63±1.04 | 1.96±0.50 |
| CuS_V_ | 6.78±1.23 | 2.13±0.78 |
| CuS_V_+NIR | 5.27±1.14 | 2.21±0.98 |

Data are presented as the mean ± SD.
